# Trametinib improves Treg selectivity of anti-CCR4 antibody by regulating CCR4 expression in CTLs in oral squamous cell carcinoma

**DOI:** 10.1038/s41598-022-22773-1

**Published:** 2022-12-15

**Authors:** Shoya Ono, Susumu Suzuki, Yutaro Kondo, Ikuko Okubo, Mitsuo Goto, Tetsuya Ogawa, Hidefumi Kato, Hideaki Ito, Taishi Takahara, Akira Satou, Toyonori Tsuzuki, Kazuhiro Yoshikawa, Toru Nagao, Ryuzo Ueda

**Affiliations:** 1 Department of Maxillofacial Surgery, Aichi Gakuin University School of Dentistry, Nagoya, 464-8651 Japan; 2 Research Creation Support Center, Aichi Medical University, Nagakute, 480-1195 Japan; 3 Department of Tumor Immunology, Aichi Medical University School of Medicine, Nagakute, 480-1195 Japan; 4 Department of Otorhinolaryngology, Aichi Medical University School of Medicine, Nagakute, 480-1195 Japan; 5 Department of Transfusion Medicine and Cell Therapy Center, Aichi Medical University Hospital, Nagakute, 480-1195 Japan; 6 Department of Pathology, Aichi Medical University School of Medicine, Nagakute, 480-1195 Japan; 7 Department of Surgical Pathology, Aichi Medical University Hospital, Nagakute, 480-1195 Japan

**Keywords:** Cancer, Immunology, Biomarkers, Oncology

## Abstract

Regulatory T-cells (Tregs) play a major role in suppressing anti-tumor immune responses. Mogamulizumab, an anti-CC chemokine receptor type 4 (CCR4) monoclonal antibody, depletes effector Tregs (eTregs). However, the clinical efficacy of mogamulizumab was limited in phase Ia/Ib studies for solid tumors (NCT01929486); the finding suggests that mogamulizumab may also deplete beneficial CCR4^+^CD8^+^ T-cells in patients. Therefore, we focused on CTLs and aimed to identify a way to protect CCR4^+^ CTLs. Here, we evaluated the association of CCR4 expression in cytotoxic T-lymphocytes (CTLs) with antigen and cytokine stimulations and kinase inhibition using cytomegalovirus antigen instead of tumor antigen. CCR4 expression in CTLs was induced by antigen stimulation (mean 3.14–29.0%), enhanced by transforming growth factor-β1 (TGF-β1) (mean 29.0–51.2%), and downregulated by trametinib with (mean 51.2–11.4%) or without TGF-β1 treatment (mean 29.0–6.98%). Phosphorylation of ERK in CD8^+^ T-cells was suppressed by trametinib. Regarding the effect on immunological function of CTL, trametinib reduced cytokine production but not affected cytotoxicity. Importantly, trametinib alleviated CTL reduction by anti-CCR4 antibody without affecting eTreg depletion because CCR4 expression in eTregs was not downregulated. In conclusion, combination therapy with trametinib may improve the clinical efficacy of mogamulizumab.

## Introduction

Immune checkpoint blockade therapy has revolutionized cancer immunotherapy. Nevertheless, most patients do not receive any clinical benefit. Regulatory T-cells (Tregs) play a major role in suppressing anti-tumor immune responses^1^. Additionally, the efficacy of anti-programmed cell death 1 (PD-1) therapy is affected by the effector Treg (eTreg) status^2,3^. Therefore, Treg depletion therapies have gained increasing research interest^4–6^.

In head and neck squamous cell carcinoma (HNSCC), anti-PD-1 therapy is the standard therapy for patients with recurrence or metastasis. However, this treatment has a median overall survival of less than 1 year^7,8^. Therefore, several clinical trials are being conducted to determine candidate drugs for combination therapy^9^. HNSCC demonstrates the highest Treg infiltration of any cancer^10^. The correlation between the expression of Forkhead Box P3 protein (FoxP3), a marker for Tregs, and HNSCC prognosis is controversial^10,11^. However, CD45RA^-^FoxP3^high^ eTregs with immunosuppressive activity within the tumors have been shown to correlate with poor prognosis^12,13^. In our previous report on HNSCC, eTregs were highly infiltrated in the tumor, and tumor-infiltrating eTregs and CD8^+^ T-cells exhibited high PD-1 expression^14^. These studies suggest the potential of depleted eTregs in immunotherapies.

Mogamulizumab, an anti-CC chemokine receptor type 4 (CCR4) monoclonal antibody (mAb), was developed to treat adult T-cell leukemia/lymphoma^15^. Since CCR4 is highly expressed on eTregs, but rarely on naive Tregs, mogamulizumab selectively depletes eTregs while maintaining naive Tregs, which are important for autoimmune suppression^1,16^; therefore, mogamulizumab is well tolerated^17^. In phase Ia/Ib studies for patients with refractory or advanced CCR4 negative solid tumors, mogamulizumab has been shown to deplete eTregs in the peripheral blood. However, in these trials, only 1 of 35 patient confirmed objective response with response evaluation criteria in solid tumors (RECIST)^18,19^. Moreover, mogamulizumab did not enhance the efficacy of checkpoint inhibitors when combined^20–22^. Since mogamulizumab has not been shown enough evidence to deplete eTregs in the tumor microenvironment, incomplete depletion of eTregs may be the cause of treatment failure. Furthermore, other cell populations expressing CCR4, especially central memory CD8^+^ T-cells (CD8^+^ TCMs), have been shown to be reduced^23^. Another potential problem is that approximately 20% of CD8^+^ tumor-infiltrating lymphocytes (TILs) express CCR4 in HNSCC patients^14^. This finding suggests that depletion of tumor-infiltrating eTregs by mogamulizumab will reduce CCR4^+^CD8^+^ TILs. Therefore, we hypothesized that protecting cytotoxic T-lymphocytes (CTLs) from antibody-dependent cellular cytotoxicity (ADCC) can overcome this potential disadvantage of anti-CCR4-Treg depletion therapy. To test this hypothesis, we aimed to downregulate CCR4 expression in CTLs without altering CCR4 expression in eTregs.

In this study, cytomegalovirus (CMV) antigen was used instead of tumor antigen to evaluate the association between antigen stimulation and CCR4 expression. We showed that CCR4 expression in CTLs is induced by T-cell receptor (TCR) signaling, with supplemental enhancement by interleukin (IL)-2, IL-12, IL-15, and transforming growth factor-beta (TGF-β1). Based on these results, we explored the downstream pathways of the TCR and cytokine receptors involved in CCR4 expression, as reported in previous studies^24–27^. Among these pathways, the MEK-ERK pathway was a common pathway except for IL-12.

Based on these studies, we tested several kinase inhibitors by selected targets that regulate cytokine signaling or MEK-ERK pathway. The results demonstrated that CCR4 expression in CTLs is strongly suppressed by trametinib, a MEK1/2 inhibitor. Therefore, in this study, we investigated the potential efficacy of trametinib as an effective agent to improve the selectivity of anti-CCR4 mAb to Tregs.

## Results

### CCR4^+^CD8^+^ T-cells are present in the tumor microenvironment

The localization of CCR4^+^CD8^+^ TILs and the expression of *CCR4* mRNA in CD8^+^ TILs had not been explored. Therefore, in this study, to examine and confirm the localization of CCR4^+^CD8^+^ T-cells in the tumor microenvironment, multifluorescence immunohistochemistry (MF-IHC) was performed. CCR4^+^CD3^+^CD8^+^ cells were identified in the stroma and tumor nest of patients with oral squamous cell carcinoma (OSCC) (Supplementary Fig. S1, white arrow). Furthermore, we also analyzed single cell RNA-sequencing (scRNA-seq) data. In TILs from the primary tumor site in five patients with OSCC, 4.19% of CD8^+^ T-cells and 11.4% of CD4^+^ T-cells expressed *CCR4* (Supplementary Fig. S2). However, immune-related mRNA expression in CD8^+^ T-cells was not characterized by *CCR4* expression.

### CCR4 expression in CTLs is induced by TCR and cytokine signaling

**Figure 1:**
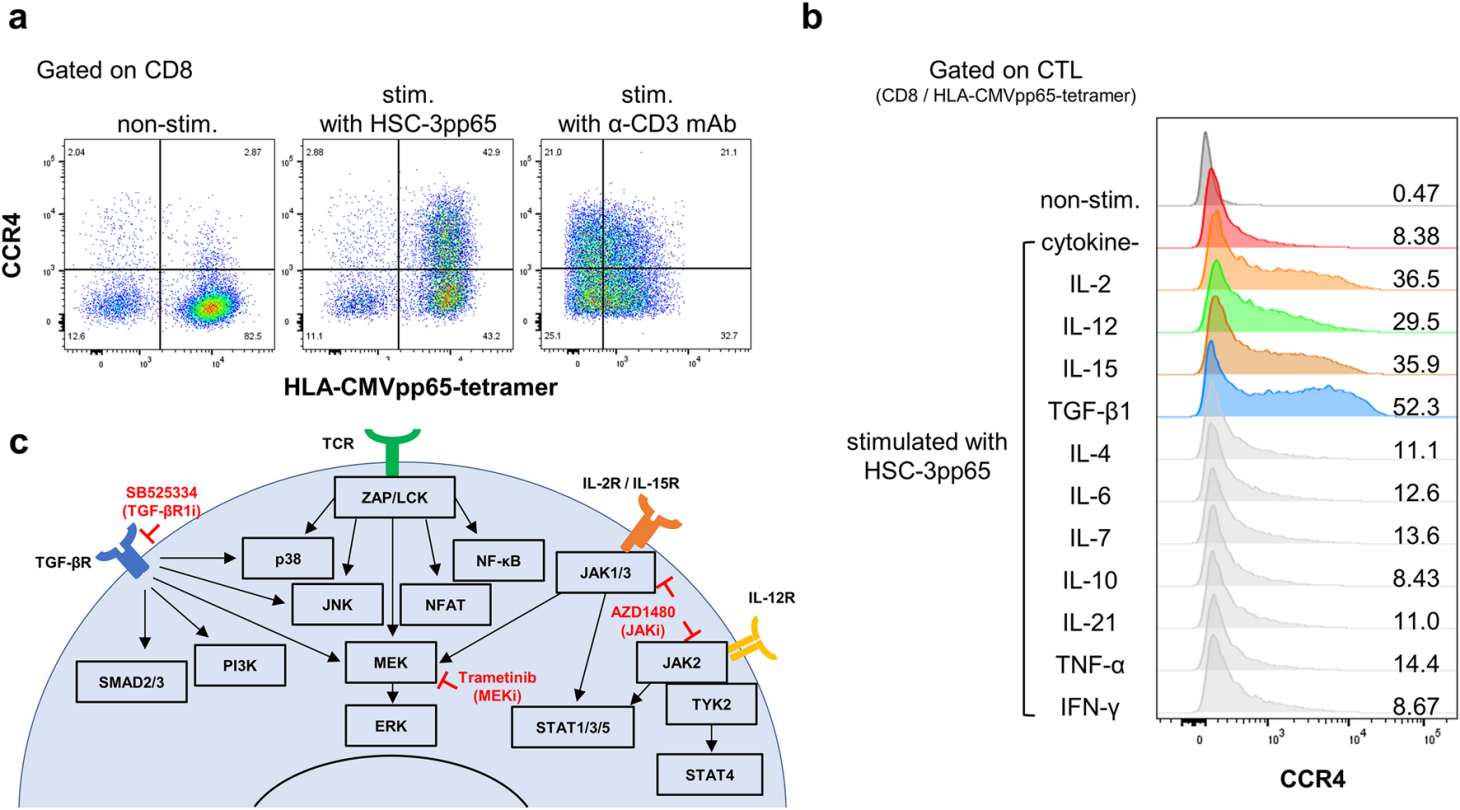
CCR4 expression in CTLs was induced by TCR and cytokine signaling. CMV-CTLs derived from PBMC of HD1 were stimulated with HSC-3pp65 cells or stimulated by immobilized anti-CD3 mAb. On day 2, flow cytometry was performed. CCR4 expression in CMV-CTLs without cytokines (**a**) and with (**b**) are shown. The receptors required for CCR4 expression obtained from the results in (**a,b**), their signaling pathways, the inhibitors used for the experiments in this study, and the sites of action are shown below (**c**). *CCR4* CC chemokine receptor type 4, *TCR* T-cell receptor, *CMV* Cytomegalovirus, *CTL* cytotoxic T-lymphocyte, *PBMC* peripheral blood mononuclear cells, *HD* healthy donor, *stim.* stimulated, *non-stim.* non-stimulated, *IL* interleukin, *IFN-γ* interferon gamma, *TNF-α* tumor necrosis factor-alpha.

We explored potential pathways to identify the candidate drug regulating CCR4 expression in CTLs. When CMVpp65-specific CTLs (CMV-CTLs) were stimulated with CMV-pp65 transfected with HSC-3 (HSC-3pp65), only a subset of CMV-CTLs showed CCR4 expression (Fig. 1a). When stimulated with the anti-CD3 mAb, CD8^+^ T-cell fraction expressed CCR4 regardless of antigen specificity. Supplementation with interleukin (IL)-2, IL-12, IL-15, or transforming growth factor-beta (TGF-β1) increased CCR4 expression in CMV-CTLs (Fig. 1b). These results demonstrated that CCR4 expression in CTLs is induced by T-cell receptor (TCR) signaling, with supplemental enhancement by IL-2, IL-12, IL-15, and TGF-β1. Based on these results, we explored the downstream pathways of the TCR and cytokine receptors involved in CCR4 expression, as reported in previous studies (Fig. 1c). Briefly, TCR signaling involved the MEK-ERK, JNK, p38, NF-κB, NFAT, and other pathways^24^. IL-2, IL-12, and IL-15 signaling use JAK-STAT as their dominant pathway^25,26^. IL-2 and IL-15 signaling also use the MEK-ERK pathway. The SMAD pathway was dominant in TGF-β signaling, but non-canonical pathways such as the MEK-ERK, PI3K-AKT, JNK, and p38 pathways were also found to be involved^27^.

### CCR4 expression in CTLs is induced by TCR stimulation, enhanced by TGF-β1, and suppressed by trametinib

**Figure 2:**
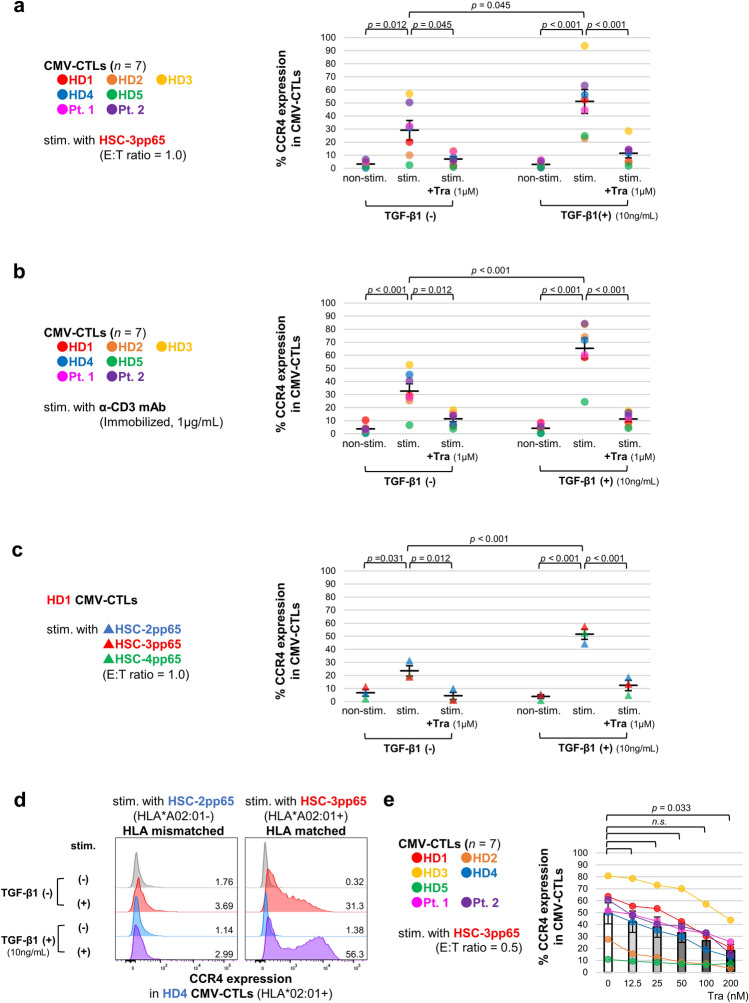
CCR4 expression in CTLs was induced by antigen-specific TCR stimulation, enhanced by TGF-β1, and suppressed by trametinib. CMV-CTLs were stimulated with HSC-2pp65, HSC-3pp65, HSC-4pp65, and immobilized anti-CD3 mAb. The E:T ratio was adjusted to 1.0 (**a**,**c**,**d**) and 0.5 (**e**). 10 ng/mL TGF-β1 and 1 μmol/L (**a–d**) or serial concentration (**e**) trametinib were added. CCR4 expression in CMV-CTLs was evaluated by flow cytometry on day 2. CCR4 expression in CTLs stimulated by HSC-3pp65 ((**a**), *n* = 7, independent donors), by anti-CD3 mAb ((**b**), *n* = 7, independent donors), by three different OSCC cell lines ((**c**), *n* = 3, independent cell lines) are shown. HLA*A02:01-restricted CMV-CTL derived from HD4 was co-cultured with HLA-mismatched HSC-2pp65 and HLA-matched HSC-3pp65. The respective CCR4 expression is shown in (**d**). Concentration-dependent effects of trametinib to downregulate CCR4 expression in CTLs co-cultured with HSC-3pp65 without TGF-β1 are shown ((**e**), *n* = 7, independent donors). Error bars indicate standard errors. Statistical analysis was performed using Tukey’s multiple comparison test (**a**–**c**) or Two-sided Dunnett’s test (**e**), and *p* < 0.05 was considered significant. Details of CMV-CTL donors are shown in Supplementary Table S1. *Pt.* patient, *E:T ratio* effector to target ratio, *Tra* trametinib, *mAb* monoclonal antibody, *nM* nmol/L, *n.s.* not significant, *HLA* human leukocyte antigen.

To regulate CCR4 expression in CTLs, we evaluated the effects of antigen stimulations, cytokines, and kinase inhibitors suggested to be potentially relevant to CCR4 expression. Details of CMV-CTL donors are shown in Supplementary Table S1. CMV-CTLs derived from three donors were treated with antigen stimulation, selected cytokines, and candidate kinase inhibitors (Supplementary Fig. S3). The common effects in the three donors on CCR4 expression in CTLs demonstrated that stimulation with specific-antigen or anti-CD3 mAb was essential for induction of CCR4 expression. It also showed that CCR4 expression was most strongly affected by TGF-β and MEK inhibitors, which were enhanced and suppressed, respectively. Therefore, we investigated only antigen stimulation, TGF-β1, and trametinib, a MEK inhibitor. First, we examined the effects of HSC-3pp65 stimulation on CCR4 expression (Fig. 2a). Antigen stimulation induced CCR4 expression in CMV-CTLs, including those from patients with OSCC (mean 3.14–29.0%), but there was a donor (HD5) with little expression. TGF-β1 increased CCR4 expression in CMV-CTLs (mean 29.0–51.2%), wherein trametinib inhibited CCR4 expression in CMV-CTLs with (mean 51.2–11.4%) or without TGF-β1 treatment (mean 29.0–6.98%). Furthermore, we evaluated the effects of TCR stimulation by antigens other than HSC-3pp65 (Fig. 2b,c). Stimulation with anti-CD3 mAb or other OSCC cell lines resulted in similar effects of TCR stimulation, TGF-β1, and trametinib on CCR4 expression in CTLs. Further, to test whether a specific antigen presentation process induces CCR4 expression in CMV-CTLs, we tested the CMV-CTLs derived from HD4, whose human leukocyte antigen (HLA) type was matched with HSC-3pp65 but not with HSC-2pp65. After co-culturing with the respective cell lines, only matched HLA types showed increased CCR4 expression in CTLs (Fig. 2d). Finally, we evaluated the dose-dependent effects of trametinib (Fig. 2e). We found that CCR4 expression in CMV-CTLs was progressively suppressed and was significant at 200 nmol/L.

### Phosphorylation of ERK in CD8^+^ T-cells is suppressed by trametinib

**Figure 3:**
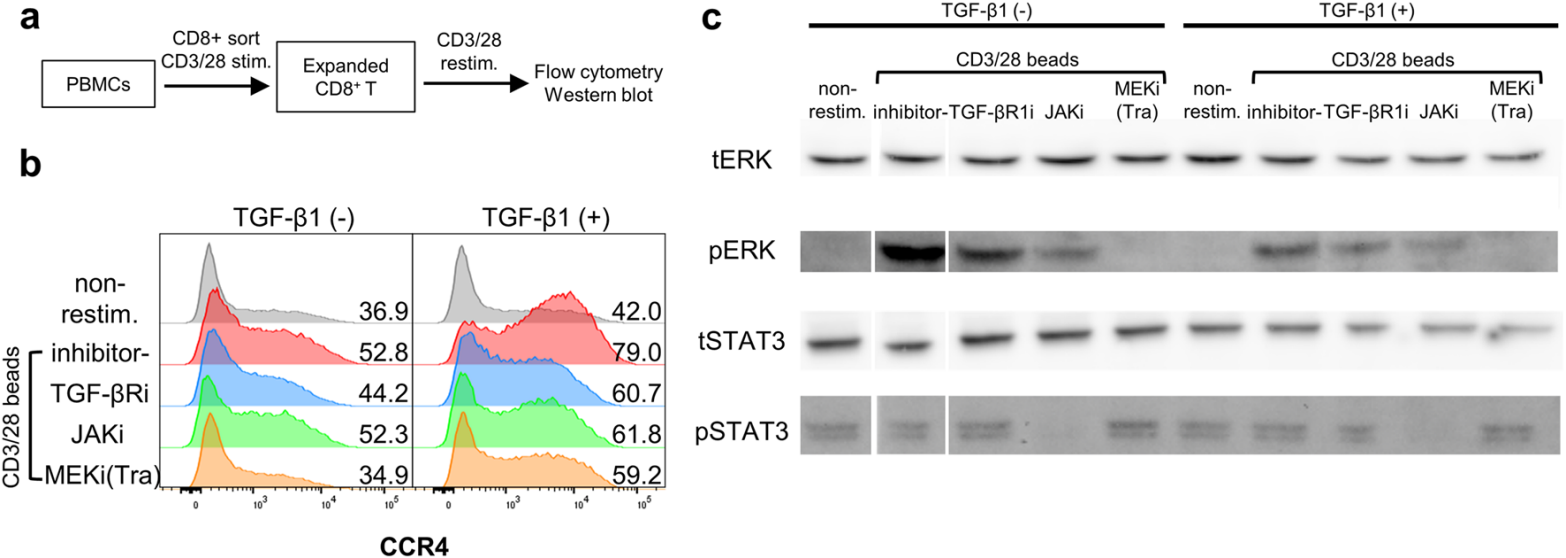
ERK in CD8^+^ T-cells was phosphorylated by CD3/28 stimulation and inhibited by trametinib. Pre-expanded CD8^+^ T-cells from HD6 were supplemented with 100 IU/mL IL-2, with or without 10 ng/mL TGF-β1, and a kinase inhibitor for 3 h. CD8^+^ T-cells were restimulated with CD3/28 beads. Experimental methods are shown in (**a**). CCR4 expression was evaluated using flow cytometry on day 2 (**b**). The same samples as in (**b**) were collected 10 min after stimulation, and phosphorylation of ERK and STAT3 was evaluated by western blotting (**c**). In the image in (**c**), the left-most and second left-most lanes are transposed. Original images are shown in Supplementary Fig. S7. *t* total, *p* phosphorylated.

To verify the reliability of the signaling pathways associated with CCR4 expression shown in Fig. 1c, phosphorylation of ERK and STAT3 was tested by western blotting. The preparation method of the sample used in the experiment is shown in Fig. 3a. Using flow cytometry, we confirmed that expanded CD8^+^ T-cells supplemented with several kinase inhibitors altered CCR4 expression in a similar trend in CMV-CTLs (Fig. 3b). However, CCR4 expression was observed without restimulation because the effects of primary stimulation remained. The effect of kinase inhibitors on CCR4 expression in CMV-CTLs was limited when TGF-β1 was added. ERK phosphorylation was induced by CD3/CD28 stimulation but not by TGF-β1 (Fig. 3c). Furthermore, MEK inhibitors strongly suppressed ERK phosphorylation but did not alter STAT3 phosphorylation. TGF-βR1 inhibitor had the least effect on ERK phosphorylation among the inhibitor tested and no effect on STAT3 phosphorylation. The JAK inhibitor suppressed STAT3 and ERK phosphorylation.

### Trametinib inhibits cytokine expression in CTL but does not affect cytotoxicity and apoptosis and has little effect on proliferation

**Figure 4:**
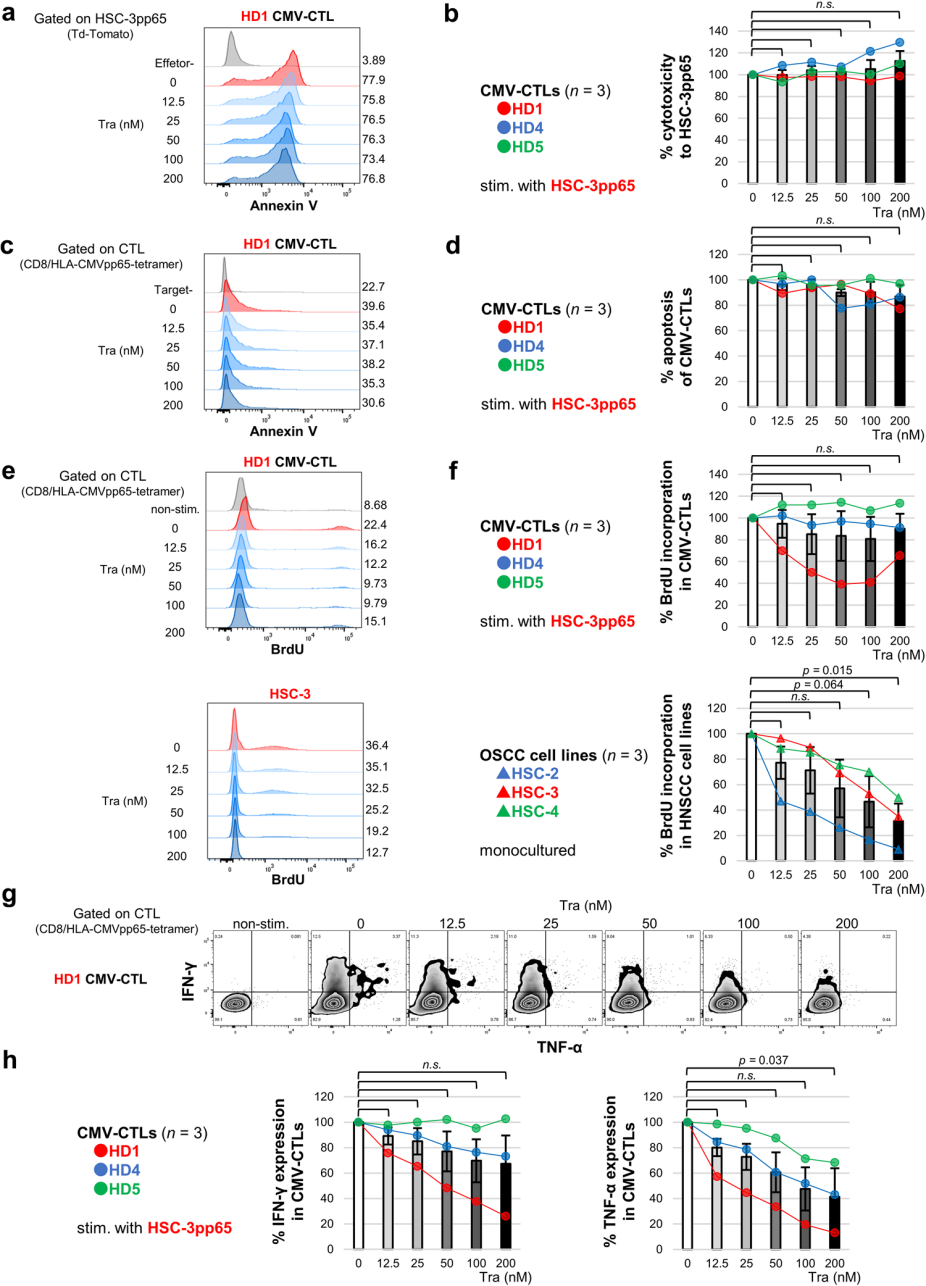
Trametinib inhibited cytokine expression in CTL, whereas cytotoxicity and apoptosis were unaffected and had little effect on proliferation. CMV-CTLs were co-cultured with HSC-3pp65 and performed flow cytometry. Cytotoxicity of CMV-CTLs was represented by the binding of Annexin V to HSC-3pp65 (**a,b**). Apoptosis of CMV-CTLs is represented by the binding of Annexin V to CMV-CTLs (**c,d**). The proliferation of CMV-CTLs and OSCC cell lines with a monoculture (**e,f**) was evaluated using the BrdU cell proliferation assay. Cytokine expression in CMV-CTLs was evaluated using intracellular IFN-γ and TNF-α staining (**g,h**). All figures are shown with a representative and summary of the result (*n* = 3, independent donors). Error bars indicate standard errors. Statistical analysis was performed using a two-sided Dunnett’s test, and *p* < 0.05 was considered significant. *OSCC* oral squamous cell carcinoma.

To evaluate the efficacy of trametinib administration for immunotherapy, the effect of trametinib on the immunological functions of CTL was examined. Cytotoxicity of CMV-CTLs and apoptosis of activated CMV-CTLs were unaffected by trametinib (Fig. 4a–d). In the 5-bromo-2′-deoxyuridine (BrdU) cell proliferation assay, no significant difference was observed in BrdU incorporation in CMV-CTLs, but one donor showed decreased incorporation (Fig. 4e,f). In contrast, BrdU incorporation in OSCC cell lines (HSC-2, HSC-3, and HSC-4) was remarkably inhibited by trametinib. Furthermore, intracellular cytokine staining showed decreased expression of tumor necrosis factor-alpha (TNF-α) by trametinib (Fig. 4g,h). No significant difference was observed in interferon-gamma (IFN-γ) expression in CMV-CTLs; however, IFN-γ expression was decreased in two of the three donors.

### Trametinib alleviates activated CTL reduction with anti-CCR4 mAb but does not affect eTreg depletion

To assess the validity of the trametinib combination, we investigated whether trametinib enhances the selectivity of anti-CCR4 mAb to Tregs. Using KM2760, an anti-CCR4 mAb with enhanced ADCC activity, we confirmed that trametinib alleviated CTL reduction without affecting eTreg depletion. For evaluation of CCR4 expression, anti-CCR4 antibody clone L291H4 was used. This clone was confirmed to have no competitive inhibition with KM2760 against the adult T-cell leukemia/lymphoma cell line MT-4, which is known to express CCR4 (Supplementary Fig. S4). In other words, the reduction in the CCR4-positive cell fraction after KM2760 treatment indicates that these cell populations have been eliminated.

**Figure 5:**
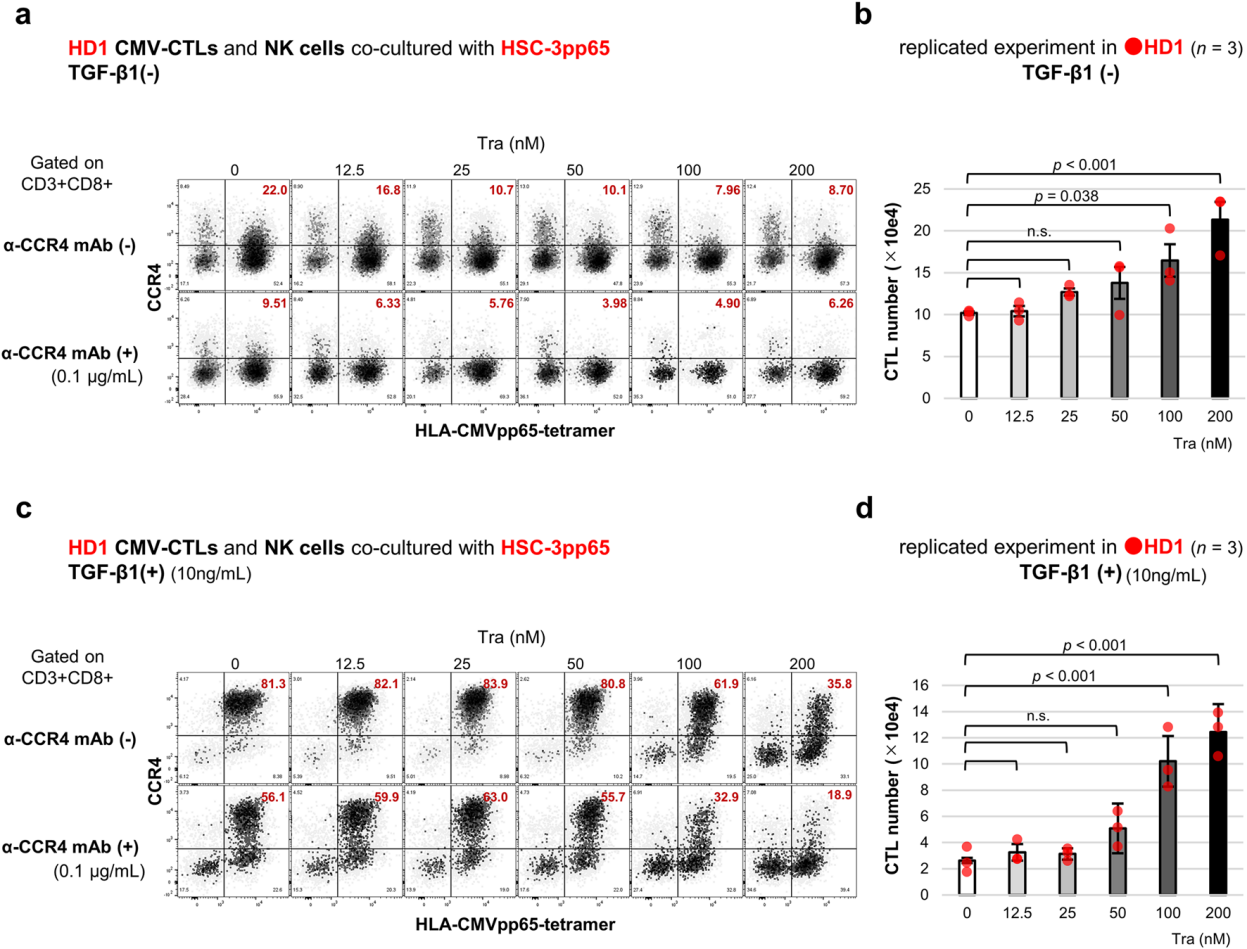
Trametinib alleviated activated CTL reduction by anti-CCR4 mAb. Lymphocytes containing expanded CMV-CTLs and NK cells derived from HD1 were co-cultured with HSC-3pp65 cells for 5 days. 0.1 μg/mL KM2760 and serial concentration of trametinib were supplemented. The results for the groups without TGF-β1 (**a,b**) and with 10 ng/mL TGF-β1 (**c,d**) are shown. HLA-CMVpp65-tetramer and CCR4 expression in CD8^+^ T-cells were evaluated using flow cytometry and are shown with a representative (**a,c**). The number of CTLs in the group treated with KM2760 is shown with a summary of results (*n* = 3, independent replicates) (**b,d**). Error bars indicate standard errors. Statistical analysis was performed using a two-sided Dunnett’s test, and *p* < 0.05 was considered significant.

A population of CTLs and NK cells used for experiments on CMV-CTL reduction with KM2760 is shown in Supplementary Fig. S5. Expression of CCR4 in CTLs was downregulated by trametinib, and CCR4^+^ CTLs was reduced by KM2760 (Fig. 5a). Trametinib alleviated CMV-CTL reduction by KM2760 through downregulation of CCR4 expression in CMV-CTLs. Compared with the non-trametinib group (mean CTL number = 10.2 × 10e^4^), the number of CTLs was higher in the 100 nmol/L trametinib (mean CTL number = 16.4 × 10e^4^, *p* = 0.038) and 200 nmol/L trametinib groups (mean CTL number = 21.3 × 10e^4^, *p* < 0.001) (Fig. 5b). Conditions with the addition of TGF-β1 were also examined. CCR4 expression was more pronounced; however, the trend of change was the same as that observed in the absence of TGF-β1 (Fig. 5c). The difference in the number of CTLs was greater in the presence than in the absence of TGF-β1. Compared with the non-trametinib group (mean CTL number = 2.63 × 10e^4^), the number of CTLs was increased in the 100 nmol/L trametinib (mean CTL number = 10.2 × 10e^4^, *p* < 0.001) and 200 nmol/L trametinib groups (mean CTL number = 12.4 × 10e^4^, *p* < 0.001) (Fig. 5d).

**Figure 6:**
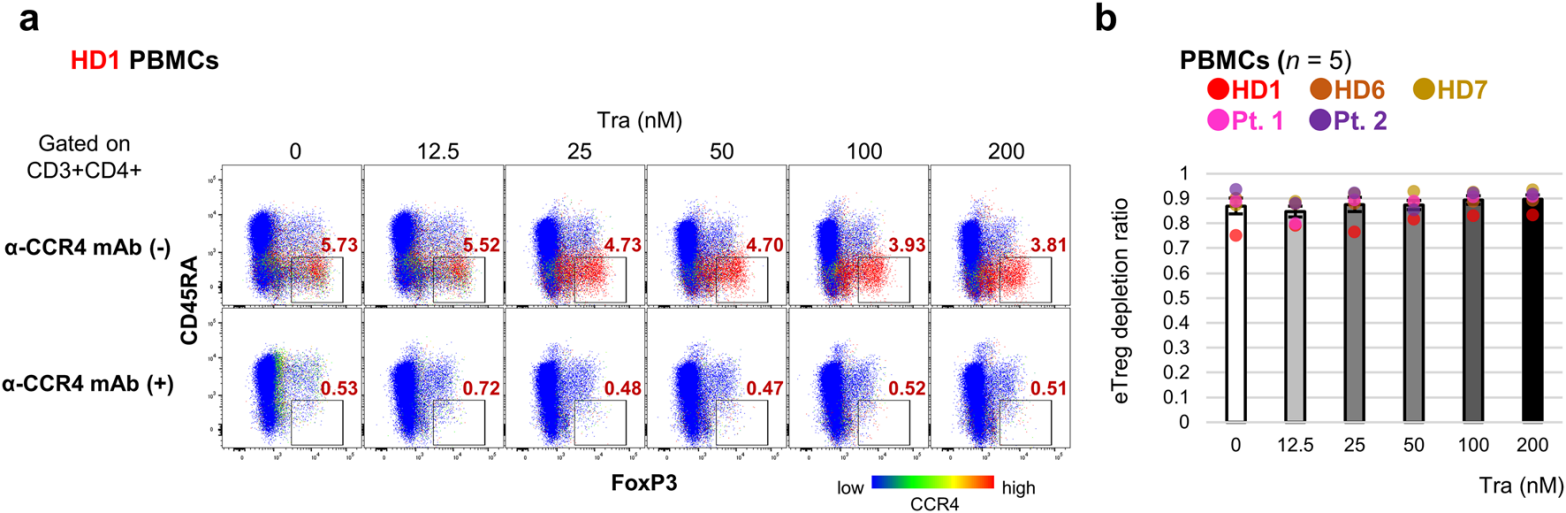
Trametinib did not affect eTreg depletion by anti-CCR4 mAb. PBMCs were incubated with 0.1 μg/mL KM2760 for 7 days and subjected to flow cytometry. CD45RA and FoxP3 expression in CD4^+^ T-cells is shown with a representative, and the intensity of CCR4 expression is presented in the heat map (**a**). The percentage of eTregs indicated by CD4^+^CD45RA^-^FoxP3^high^ is displayed in the figure. The depletion ratio of eTregs in the KM2760 group is shown with a summary of the result (*n* = 5, independent donors) (**b**). Error bars indicate standard errors.

In the eTreg depletion assay, KM2760 depleted all CCR4^+^ cells in the peripheral blood mononuclear cells (PBMCs). CD4^+^CD45RA^-^FoxP3^high^ eTregs highly express CCR4 (Fig. 6a). Trametinib did not suppress CCR4 expression in eTregs. As a result, eTregs were almost removed. The eTreg depletion ratio was high (> 0.8), and no difference was observed among different trametinib concentrations (Fig. 6b). Such eTreg depletion was observed in both healthy donors and patients. The PBMCs used in this assay contained CCR4^+^CD8^+^ T-cells. CD8^+^ T-cells already expressing CCR4 did not downregulate it by trametinib (Supplementary Fig. S6a) and were completely depleted by KM2760, regardless of trametinib administration (Supplementary Fig. S6b). CCR4^+^CD8^+^ T-cell reduction was also observed in both healthy donors and patients.

## Discussion

Treg depletion therapy is expected to be the next novel tumor immunotherapy, and several clinical trials targeting Tregs using mAbs, such as CD25 (daclizumab)^28^ and CCR4 (mogamulizumab)^18^, have been conducted. However, the clinical benefits of Treg depletion are limited. One of the hypothesized reasons for these unexpected results is the expression of these target molecules on conventional T-cells, including CD8^+^ T-cells, besides Tregs. Ipilimumab, an anti-CTLA-4 mAb known to function as an immune checkpoint inhibitor, also induces ADCC in Tregs^29–31^. However, CTLA-4 is also expressed on activated effector T-cells. Thus, improving the targeting specificity to Tregs is necessary for successful Treg depletion therapy.

There have been several reports on CCR4 expression in CD8^+^ T-cells. Peripheral CCR4^+^CD8^+^T-cells are predominantly memory cells^32^. Peripheral blood samples obtained from a clinical trial with mogamulizumab showed depletion of CD8^+^ TCM^23^. Within the tumor microenvironment, CCR4 is also expressed on CD8^+^ TILs in HNSCC by flow cytometry^14^. In this study, using MF-IHC, we confirmed that CCR4^+^CD8^+^ T-cells infiltrated OSCC tissues (Supplementary Fig. S1). In addition, bioinformatic analysis of scRNA-seq data showed that several CD8^+^ T-cells transcribed CCR4 (Supplementary Fig. S2). Since CD8^+^ TILs are mostly effectors, CCR4^+^CD8^+^ TILs are not considered TCMs. Most CCR4^+^CD8^+^ T-cells are distributed along the periphery of the tumor nest, and some infiltrate the tumor nest. T-cells might be activated in these areas; therefore, CCR4 expression in CD8^+^ TILs is thought to be related to activation. We investigated this hypothesis in vitro. We used the CMVpp65 antigen as a cancer antigen model because it is difficult to prepare enough real tumor antigen-specific CTLs. Since the CMVpp65 antigen is a highly immunogenic foreign antigen, the binding affinity of TCR on CMV-CTLs to the peptide-HLA complex is high. However, neoantigens and oncogenic papilloma viral antigens, which are highly immunogenic and play an important role in anti-tumor immunity for OSCCs, have similar characteristics to CMVpp65 antigen.

CCR4 expression in CTLs was induced by TCR stimulation (Fig. 2). When TCR signaling was transduced, cytokine signaling enhanced CCR4 expression. These findings suggest that CCR4^+^CD8^+^ T-cells are antigen-specific CTLs with activated TCR signaling. Therefore, CCR4^+^CD8^+^ TILs should be protected from ADCC by mogamulizumab to augment anti-tumor immunity. Trametinib inhibited the phosphorylation of MEK downstream of TCR and markedly reduced CCR4 expression in CTLs, which resulted in the alleviation of CTL reduction by ADCC when treated with anti-CCR4 mAb KM2760 (Figs. 2, 3, 5). However, a donor (HD5) had difficulty inducing CCR4 expression in CTLs by antigen stimulation alone (Fig. 2a). Therefore, predicting CCR4 expression in CTLs is important from the perspective of precision medicine.

The effect of trametinib to regulate CCR4 expression in CTLs differed from in eTregs and peripheral CCR4^+^CD8^+^ T-cells (Figs. 5, Supplementary Fig. S6). In our experiments, CTLs expressing CCR4 after antigen stimulation were treated with trametinib before CCR4 expression. In contrast, eTregs and peripheral CCR4^+^CD8^+^ T-cells, expressing CCR4 constantly, were treated with trametinib after CCR4 expression. Our results suggest that the different effects of trametinib on CCR4 expression in CTLs, eTregs, and peripheral CCR4^+^CD8^+^ T-cells depend on the timing of CCR4 expression. Treg depletion by mogamulizumab is expected to result in CTL activation in vivo. Therefore, administration of trametinib prior to mogamulizumab administration may improve target selectivity to eTregs in the tumor microenvironment. The treatment schedule for therapeutic application will be determined by the pharmacokinetics/pharmacodynamics of both trametinib and mogamulizumab in the tumor microenvironment. Thus, it is necessary to establish an in vivo model in which mogamulizumab can exerts ADCC activity in tumor microenvironment to determine the treatment schedule in the future. The protection of CCR4^+^CD8^+^ T-cells in peripheral blood must be considered separately from this discussion. However, the selectivity of mogamulizumab to Tregs in peripheral CCR4^+^CD8^+^ T-cells might increase by adjusting the concentration in the blood because CD8^+^ T-cell susceptibility to mogamulizumab is lower than that of eTregs in vitro^23^.

Since TGF-β1 strongly enhanced CCR4 expression in CTLs (Fig. 2), we initially hypothesized that the MEK-ERK pathway was involved. However, contrary to expectations, supplementation with TGF-β1 did not enhance ERK phosphorylation in CD8^+^ T-cells (Fig. 3). In this study, we failed to clarify the pathway by which TGF-β1 enhances CCR4 expression. Nevertheless, TGF-β1 signaling is undoubtedly one of the most promising targets. In addition, *TGFB1* mRNA is abundantly distributed in the invasive front of OSCCs where the infiltration by T-cells is high, and inhibition of TGF-β1 signaling restore CTL function^33^. Taken together, TGF-β1 is a therapeutic target not only for the downregulation of CCR4 expression in CTLs but also for restoring CTL function. Nevertheless, further investigation is needed to clarify the signaling pathway through which TGF-β1 affects T-cell function.

Trametinib is an allosteric inhibitor with very high selectivity for MEK1/2^34^. The main targets of trametinib are malignant melanoma and non-small cell lung cancer with RAF/RAS mutation^35^. Trametinib is also expected to have an anti-tumor effect in OSCC. Despite infrequent RAS/RAF mutations in HNSCC^36,37^, phosphorylation of ERK1/2 occurs in more than 90% of HNSCC cases^38,39^. In a neoadjuvant clinical trial of trametinib for OSCC, 65% of patients showed tumor shrinkage, and 54% showed downstaging^40^. In the present study, trametinib treatment did not affect the cytotoxicity or apoptosis of the CMV-CTLs (Fig. 4a–d). Furthermore, trametinib treatment markedly inhibited the proliferation of tumor cell lines but had little effect on the proliferation of CMV-CTLs (Fig. 4e,f). However, trametinib treatment inhibited cytokine production in CTLs (Fig. 4g,h). Previous reports have similarly shown decreased cytokine production following trametinib treatment in vitro but increased T-cell infiltration and enhanced anti-tumor immunity in vivo^41^. This could be because of improved effector to target (E:T) ratio due to selective inhibition of tumor cell growth and decreased myeloid-derived suppresser cells^42^. Our study evaluated the effect of trametinib on individual immunological function and identified the suppression of cytokine production as a potential drawback. However, anti-tumor immunity is regulated by a more complex mechanism in vivo, and trametinib enhances anti-tumor immunity comprehensively. The findings of the previous and present studies suggest the promising outcome of concomitant use of trametinib with Treg-targeted immunotherapy using mogamulizumab. Although the maximum plasma concentration of the approved dose of trametinib (2 mg orally) is 14–32.9 ng/mL^43^ (~ 22.8–53.5 nmol/L), a higher concentration was required for a stronger effect on the downregulation of CCR4 in this study. Therefore, decreasing the plasma concentration of trametinib is important for clinical application with minimal side effects. A possible strategy to increase the local concentration, as in local/intra-arterial injections, should be considered.

This study had several major limitations. First, little clinical evidence indicates that mogamulizumab reduces the number of eTregs and CCR4^+^CD8^+^ TILs. Clinical trials in neoadjuvant settings may help to prove this hypothesis. Second, antigen specificity of CCR4^+^CD8^+^ TILs has not yet been demonstrated. In other words, this in vitro study does not ensure extrapolation to the human tumor microenvironment. This study was performed with only a small number of immune cell donors and with surrogate non-tumor antigen systems. We should collect CCR4^+^CD8^+^ TILs and evaluate their response to tumor antigens. Third, the detailed mechanism of CCR4 expression in CTLs remains unclear. Elucidation of this mechanism provides a benchmark for determining the indications for trametinib administration in mogamulizumab treatment.

In conclusion, CCR4^+^ CTLs show antigen specificity in an in vitro model. Trametinib alleviates anti-CCR4 mAb-induced CTL reduction by downregulating CCR4 expression in CTLs through modulation of TCR signaling. In contrast, trametinib does not affect eTreg depletion. Overall, the study suggests trametinib as a promising adjuvant for mogamulizumab to alleviate CTL reduction.

## Methods

### Cell culture

OSCC cell lines, HSC-2, HSC-3, and HSC-4, were maintained in DMEM (FUJIFILM Wako Pure Chemical Corporation, Osaka, Japan) supplemented with 10% fetal bovine serum (FBS; HyClone Laboratories, Inc., South Logan, UT, USA) and 1% penicillin–streptomycin (Life Technologies, Carlsbad, CA, USA) in 75 mL flasks at 37 °C in 5% CO_2_ humidified air. HSC-2pp65, HSC-3pp65, and HSC-4pp65 cells transfected with CMVpp65 antigen and tdTomato in HSC-2, HSC-3, and HSC-4 cell lines were prepared as previously described^44^. Afterward, 250 µg/mL G418 (FUJIFILM Wako Pure Chemical Corporation) was added to maintain these cell lines concentration.

### Clinical sample

For MF-IHC, patients with OSCC who had undergone surgical resection without neoadjuvant therapy at the Department of Maxillofacial Surgery, Aichi Gakuin University Dental Hospital, were selected. Peripheral blood was collected from previously untreated patients with clinical stage IV OSCC according to the eighth edition of the TNM classification at the Department of Otorhinolaryngology-Head and Neck Surgery, Aichi Medical University Hospital. Details of the donor disease status are shown in Supplementary Table S1. This study was conducted in accordance with the Declaration of Helsinki and approved by the Ethical Committee of Aichi Gakuin University (approval number: 82) and Aichi Medical University (approval numbers: 2020-H033 and 2020-H073). Donor and patient blood samples were obtained with informed consent from all study subjects.

### Induction of CMV-CTLs

HLA-A*02:01 and HLA-A*24:02 restricted CMV-CTLs were prepared according to a method reported previously^44^. PBMCs derived from healthy donors or patients were used. Healthy donors included healthy volunteers or donors for allogeneic peripheral blood stem cell transplantation, which had lost their use. PBMCs were isolated by density gradient centrifugation using a Ficoll-Paque PLUS (GE Healthcare, Chicago, IL, USA) or collected by apheresis. Details of HLA restriction and collection method of PBMCs are shown in Supplementary Table S1.

### Flow cytometry

Multicolor panels for flow cytometry are shown in Supplementary Table S2 (fluorophore-conjugated antibodies were purchased from BioLegend, San Diego, CA, USA; BD Bioscience, Franklin Lakes, NJ, USA; MBL, Tokyo, Japan). The cells were collected and washed in PBS containing 0.25% human serum albumin and 2.5 mmol/L EDTA. The cells were then stained with antibodies at 4 °C for 20 min. To analyze the CMV-CTL population, cells were stained with fluorophore-conjugated HLA-A*02:01 CMVpp65 tetramer-NLVPMVATV or HLA-A*24:02 CMVpp65 tetramer-QYDPVAALF for 10 min prior to staining with other antibodies. Flow cytometry was performed with BD LSRFortessa (BD Bioscience), and the data were analyzed using FlowJo ver.10.8.1 (BD Bioscience).

### Induction of CCR4 expression in CMV-CTLs

CMV-CTLs (1 × 10^5^ cells) were stimulated with CMVpp65 transfected OSCC cell lines or 1 μg/mL immobilized OKT-3 anti-CD3 mAb (clone OKT-3, eBioscience, San Diego, CA, USA) in serum-free Alys505N-0 medium for 2 days. Several cytokines (10 ng/mL) and kinase inhibitors (1 μmol/L) were added. The cytokines and kinase inhibitors used were as follows: IL-2, IL-4, IL-6, IL-7, IL-10, IL-12, IL-15, IL-21, TGF-β1, TGF-β3, TNF-α, IFN-γ (all from Miltenyi Biotec Inc., Bergisch Gladbach, Germany), SB525223 (TGF-β receptor 1 (TGF-βR1) inhibitor, Selleck, Houston, TX, USA), AZD1480 (JAK inhibitor, Selleck), and GSK1120212 (trametinib, a MEK1/2 inhibitor, Selleck).

### Western blotting

PBMCs from HD6 were isolated using the CD8^+^ T-cell Isolation Kit and AutoMACS (Miltenyi Biotec Inc.), according to the manufacturer’s protocol. Isolated CD8^+^ T-cells were expanded with T-cell Activation/Expansion Kit (Anti-Biotin MACSiBead Particles and biotinylated antibodies against human CD2, CD3, and CD28; Miltenyi Biotec Inc.) in Alys505-N supplemented with 5% FBS and 100 IU/mL IL-2 for 14 days. Expanded CD8^+^ T-cells were supplemented with 10 ng/mL TGF-β1 and kinase inhibitors (1 μmol/L) in an Alys505N-0 medium supplemented with 100 IU/mL IL-2 for 3 h. Treated CD8^+^ T-cells were restimulated using the T-cell Activation/Expansion Kit for 10 min. The cells were lysed with lysis buffer (1% TritonX-100, 2 mmol/L 2-mercaptoethanol, and 2 mmol/L EDTA-containing Tris-buffered salines). The cell lysates were boiled at 95 °C for 5 min in an SDS sample buffer. The samples were separated by SDS-PAGE and transferred to PVDF membranes by dry blotting. After blocking with TBS containing 5% skim milk for 1 h at room temperature, the membranes were incubated overnight with primary antibodies. Anti-Erk1/2, anti-phospho-Erk1/2, anti-STAT3, and anti-phospho-STAT3 (all from Cell Signaling Technology, Danvers, MA, USA) were used as primary antibodies at 1:4000 dilution. The membranes were washed three times with TBS and incubated with secondary antibodies for 30 min. Peroxidase polymer anti-mouse or anti-rabbit IgG (Vector Laboratories, Burlingame, CA, USA) were used as secondary antibodies at 1:400 dilution. After washing with TBS three times, the protein signals were detected using SuperSignal West Atto (Thermo Fisher Scientific, Waltham, MA, USA) and imaged using ImageQuanto LAS 500 (Cytiva, Stockholm, Sweden).

### Cytotoxicity and apoptosis assay with Annexin V

CMV-CTLs were co-cultured with or without HSC-3pp65 cells in an Alys505N-0 medium supplemented with 5% FBS, 100 IU/mL IL-2, and serial concentrations of trametinib for 24 h. The E: T ratio was adjusted to 0.5. Annexin V staining was performed as previously reported^33^. %Cytotoxicity to HSC-3pp65 and % apoptosis of CMV-CTLs were calculated using the following formula:

$$\text{\% Cytotoxicity to HSC-3pp65}=\frac{\text{\%annexin expression in tdTomato}^{+}\text{ cells with each concentration trametinib}}{\text{\% annexin expression in tdTomato }^{+}\text{ cells with 0 nM trametinib}}\times 100,$$

$$\text{\%Apoptosis of CMV-CTLs}=\frac{\text{\% annexin expression in CD8}^{+}\text{HLA CMVpp65 tetramer}^{+}\text{ cells with each concentration trametinib}}{\text{\% annexin expression in CD}}{8}^{+}\text{HLA CMVpp65 tetramer}^{+}\text{ cells with 0 nM trametinib} \times 100.$$

### Cell proliferation assay with BrdU

OSCC cell lines were maintained in serum-free DMEM for 7 days. CMV-CTLs were co-cultured with HSC-3pp65 cells in an Alys505N-0 medium supplemented with 5% FBS, 100 IU/mL IL-2, and serial concentrations of trametinib for 2 days. The E: T ratio was adjusted to 0.5. For the analysis of cell proliferation, 10 μmol/L BrdU was added to each sample and incubated for 1 h. Cell staining methods have been previously reported^33^. % BrdU incorporation was calculated using the following formula:

$$\% {\mkern 1mu} {\text{BrdU incorporation}} = \frac{{\% {\text{BrdU expression in CD8}}^{ + } {\text{ HLA CMVpp65 tetramer}}^{ + } {\text{ cells with each concentration trametinib}}}}{{\% {\mkern 1mu} {\text{BrdU expression in CD}}8^{ + } {\text{HLA CMVpp65 tetramer}}^{ + } {\text{ cells with 0 nM trametinib}}}} \times 100.$$

### Intracellular cytokine staining

CMV-CTLs were co-cultured with HSC-3pp65 cells in an Alys505N-0 medium supplemented with 5% FBS, 100 IU/mL IL-2, and serial concentrations of trametinib for 4 h. Monensin (1 μg/mL) was added during the last 2 h. The E:T ratio was adjusted to 0.5. The cells were stained with surface marker antigens and fixed with 4% formaldehyde for 20 min. After washing two times, a permeabilization buffer containing saponin at a final concentration of 0.25% was added, and cytokine staining was performed at 4 ℃ for 30 min. %Cytokine expression was calculated using the following formula:

$$\text{\% Cytokine expression}=\frac{\text{\% cytokine expression in CD}{8}^{+} {\text{HLA CMVpp65 tetramer}}^{+}\text{ cells with each concentration trametinib}}{\text{\% cytokine expression in CD}{8}^{+} {\text{HLA CMVpp65 tetramer}}^{+}\text{ cells with 0 nM trametinib}}\times 100.$$

### Effects of ADCC by KM2760 on the proliferation of CMV-CTLs

HD1 CMV-CTLs containing a large number of NK cells were used. The CMV-CTLs were co-cultured with HSC-3pp65 in an Alys505N-0 medium supplemented with 5% FBS, 100 IU/mL IL-2, and serial concentration of trametinib with or without 0.1 μg/mL KM-2760 (defucosylated chimeric anti-CCR4 IgG1 mAb; Kyowa Kirin). The E: T ratio (CMV-CTL to HSC-3pp65 ratio) was adjusted to 1.0. After 5 days, the cells were collected and subjected to flow cytometry. The CTL number was calculated using the following formula:

$$\text{CTL number}=\text{total cell}\times \text{percentage of CD}{8}^{+}\text{HLA CMVpp65 tetramer}^{+}\text{ cells}.$$

### eTreg depletion by KM2760 in PBMCs

PBMCs were cultured in an Alys505N-0 medium supplemented with 5% FBS, 100 IU/mL IL-2, and serial concentrations of trametinib, with or without 0.1 μg/mL KM2760. After 7 days, the cells were subjected to flow cytometry. FoxP3 was performed using the FoxP3/Transcription Factor Staining Buffer Set (Thermo Fisher Scientific), according to the manufacturer’s protocol. The eTreg depletion ratio and CCR4^+^CD8^+^T-cell depletion ratio were calculated using the following formula:

$$\text{eTreg depletion ratio}=1-\frac{\text{\% CD}45{\text{RA}}^{+}\text{FoxP}{3}^{\text{high}}{\text{ in CD}}{3}^{+}\text{CD}{4}^{+}\text{ cells at day }7}{\text{\% CD}45{\text{RA}}^{+}\text{FoxP}{3}^{\text{high}}{\text{ in CD}}{3}^{+}\text{CD}{4}^{+}\text{ cells at day }0},$$

$$\text{CCR}{4}^{+}\text{CD}{8}^{+}\text{T cell depletion ratio}=1-\frac{\text{\% CCR}{4}^{+}\text{ in CD}{3}^{+}\text{CD}{8}^{+}\text{ cells at day }7}{\text{\% CCR}{4}^{+}\text{ in CD}{3}^{+}\text{CD}{8}^{+}\text{ cells at day }0}.$$

### MF-IHC

Formalin-fixed, paraffin-embedded OSCC section was stained according to a method reported previously^33^. The following primary antibodies were used: CD3 (clone M4622; Spring Biosciences, Eugene, OR, USA), CD8 (clone 1A5; Biogenex, Fremont, CA, USA), FoxP3 (clone 236A/E7; Abcam, Cambridge, UK), CCR4 (clone KM2160; mouse anti-CCR4 mAb; Kyowa Kirin), and cytokeratin (clone CAM5.2; Biogenex). Nuclei were stained with DAPI. Images were captured using Vectra (PerkinElmer, Waltham, MA, USA).

### Bioinformatic analysis

In scRNA-seq, the expression levels were downloaded from the Gene Expression Omnibus (August 20th, 2021 https://www.ncbi.nlm.nih.gov/geo/query/acc.cgi?acc=GSE103322). The expression levels were calculated by modifying transcripts per million (TPM) values^45^. Based on the expression levels of T-cells from the primary site of five OSCC patients, heatmap analysis was performed using IDEP.93 (October 15th, 2021 http://bioinformatics.sdstate.edu/idep/)^46^. CCR4^+^ T-cells were identified using a Z-score of > 2.

### Statistical analysis

Numerical data are presented as the mean and standard error. Statistical analysis was performed using EZR software (ver.3.6.1)^47^. A two-sided Dunnett’s or Tukey’s multiple comparison test was employed for multiple group comparisons. *p* < 0.05 was considered significant.

### Ethics declarations

This study was conducted in accordance with the Declaration of Helsinki and approved by the Ethical Committee of Aichi Gakuin University (Approval Number: 82) and Aichi Medical University (Approval Numbers: 2020-H033 and 2020-H073).

### Consent to participate

For non-personally identifiable patient samples and clinical data, we provided patients with the opportunity to refuse in an opt-out approach.

## Supplementary Information

Supplementary Information.

## Data Availability

The data will be shared on reasonable request with the corresponding author.
