# Supplementary material for: Trametinib improves Treg selectivity of anti-CCR4 antibody by regulating CCR4 expression in CTLs in oral squamous cell carcinoma
Source: Sci Rep. 2022 Dec 15;12:21678. doi: 10.1038/s41598-022-22773-1 (PMC9755268; doi:10.1038/s41598-022-22773-1)
Supplement: Supplementary file 1 — Supplementary Information. [file 41598_2022_22773_MOESM1_ESM.pdf]

Supplementary information

scientific reports (submitted in 2022) –Ono et al.

Supplementary Table S1 List of donors for PBMC isolation

Donor	Disease	PBMC isolation method	Epitopes used for CMV-CTL induction
**HD1**	None	Density gradient centrifugation	QYDPVAALF (HLA-A*24:02 restricted)
**HD2**	None	Density gradient centrifugation	NLVPMVATV (HLA-A*02:01 restricted)
**HD3**	None	Density gradient centrifugation	NLVPMVATV (HLA-A*02:01 restricted)
**HD4**	None	Apheresis (for PBSCT)	NLVPMVATV (HLA-A*02:01 restricted)
**HD5**	None	Apheresis (for PBSCT)	NLVPMVATV (HLA-A*02:01 restricted)
**HD6**	None	Density gradient centrifugation	—
**HD7**	None	Density gradient centrifugation	—
**Pt. 1**	OSCC (tongue, T4aN2bM0)	Density gradient centrifugation	NLVPMVATV (HLA-A*02:01 restricted)
**Pt. 2**	OSCC (lower gingiva, T4aN2cM0)	Density gradient centrifugation	NLVPMVATV (HLA-A*02:01 restricted)

HD: healthy donor, Pt: Patient, OSCC: oral squamous cell carcinoma, CMV-CTL: cytomegalovirus specific cytotoxic T-lymphocyte, HLA: human leukocyte antigen, PBSCT: peripheral blood stem cell transplantation

Supplementary Table S2 Multicolor panels for flow cytometry.

antibody / molecule	conjugate	clone	manufacture
CCR4 expression			
HLA-CMVpp65-tetramer	APC	-	MBL
CD8	APC-Cy7	RPA-T8	BioLegend
CCR4	BV421	L291H4	BioLegend
BrdU cell proliferation assay			
HLA-CMVpp65-tetramer	APC	-	MBL
CD8	APC-Cy7	RPA-T8	BioLegend
CCR4	BV421	L291H4	BioLegend
BrdU	FITC	3D4	BioLegend
Annexin V cytotoxicity/apoptosis assay			
HLA-CMVpp65-tetramer	APC	-	MBL
CD8	APC-Cy7	RPA-T8	BioLegend
CCR4	BV421	L291H4	BioLegend
Annexin V	FITC	-	BioLegend
Intracellular cytokine staining			
HLA-CMVpp65-tetramer	PE	-	MBL
CD8	APC-Cy7	RPA-T8	BioLegend
CCR4	BV421	L291H4	BioLegend
IFN-γ	FITC	4S.B3	eBioscience
TNF-α	APC	Mab11	eBioscience
Effects of ADCC by KM2760 on the proliferation of CMV-CTLs			
HLA-CMVpp65-tetramer	APC	-	MBL
CD3	FITC	UCHT1	BioLegend
CD8	APC-Cy7	RPA-T8	BioLegend
CD16	BV480	3G8	BD Bioscience
CD56	PE	5.1H11	BioLegend
CCR4	BV421	L291H4	BioLegend
eTreg depletion by KM2760 in PBMCs			
CD3	BUV737	UCHT1	BD Bioscience
CD4	APC	RPA-T4	BioLegend
CD8	BUV395	RPA-T8	BD Bioscience
CD16	BV480	3G8	BD Bioscience
CD45RA	APC-Cy7	HI100	BioLegend
CD56	PE	5.1H11	BioLegend
CCR4	BV421	L291H4	BioLegend
FoxP3	Alexa488	236A/E7	BD Bioscience

CCR4: CC chemokine receptor type 4, BrdU: 5-bromo-2’-deoxyuridine, ADCC: antibody-dependent cellular cytotoxicity, eTreg: effector regulatory T-cell, PBMC: peripheral blood mononuclear cells, FoxP3: Forkhead Box P3 protein,

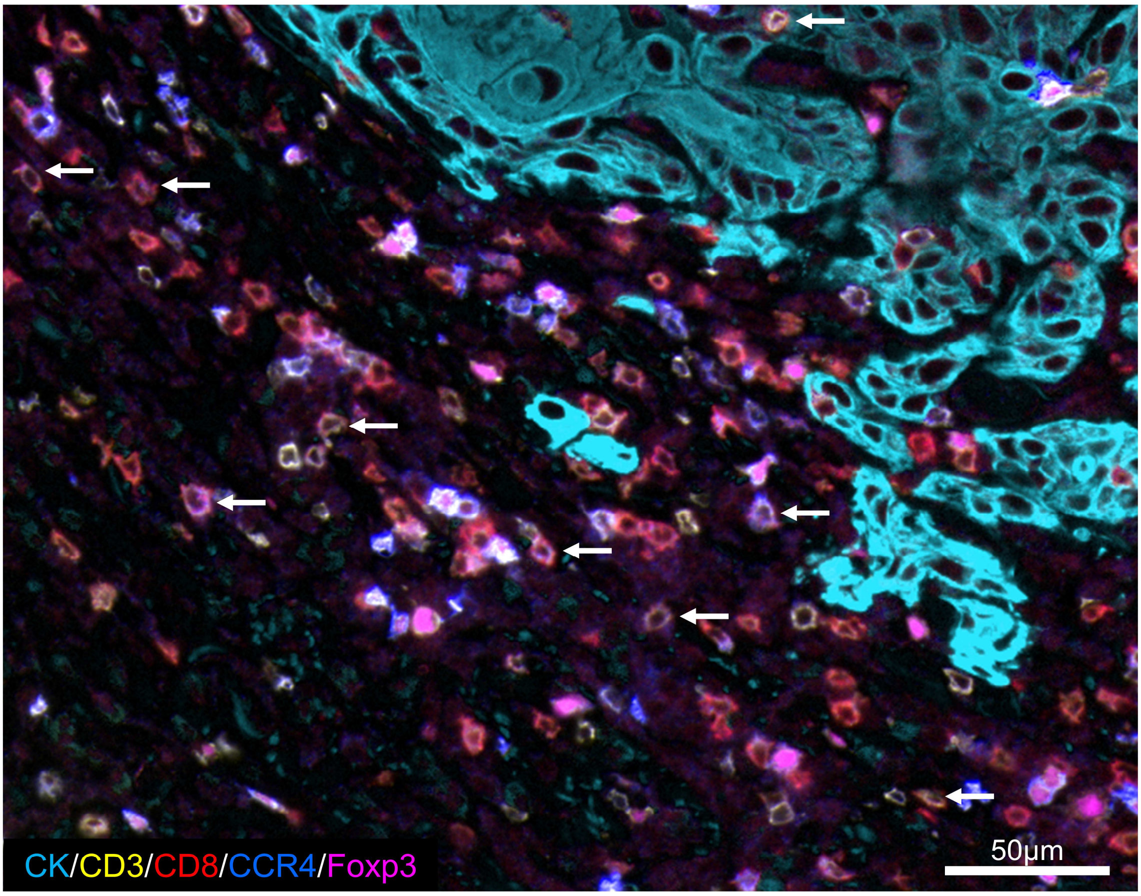

Supplementary Figure S1 CCR4$^+$CD8$^+$ T-cells was detected in OSCC microenvironment by MF-IHC. Representative images of MF-IHC at the primary site of OSCC (×200 magnification). White arrows indicate CCR4$^+$CD8$^+$ T-cells. OSCC: oral squamous cell carcinoma, MF-IHC: multifluorescence immunohistochemistry

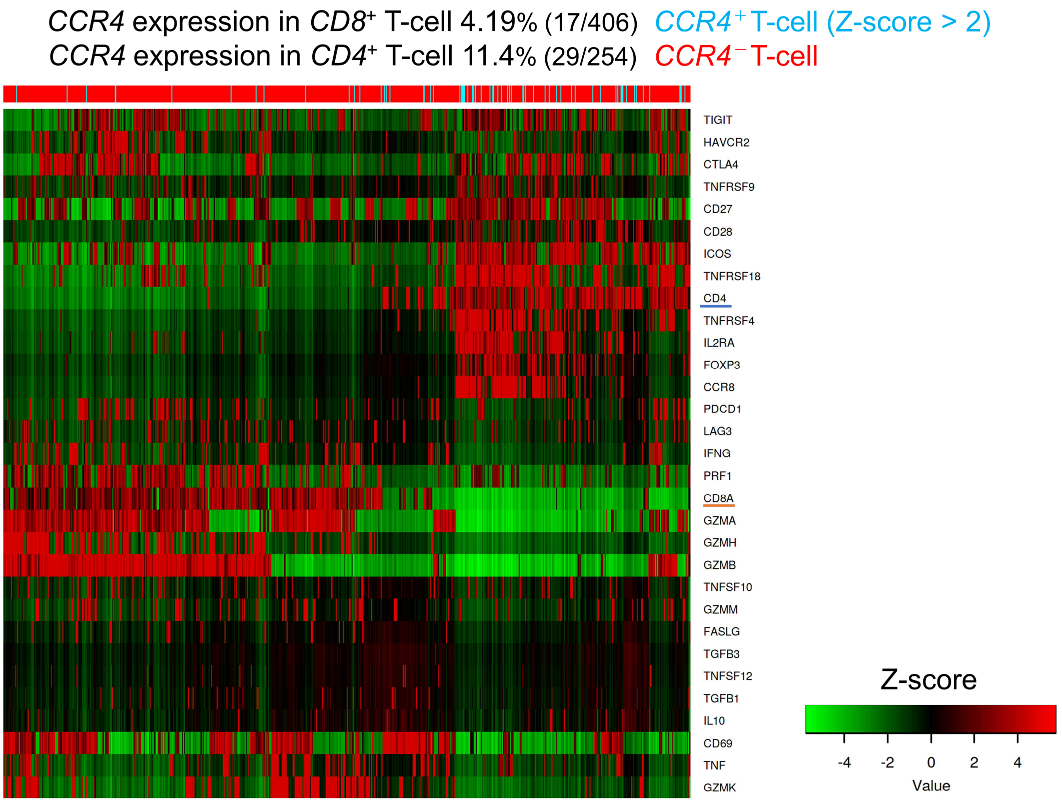

Supplementary Figure S2 A part of the *CD8*$^{+}$ T-cell population were transcribed *CCR4* mRNA in OSCC primary site. Heatmap analysis based on the expression levels calculated by scRNA-seq raw data of T-cells from the primary site of five OSCC patients. *CCR4*$^{+}$ T-cell were identified with a Z-score > 2. scRNA-seq: single cell RNA-sequencing

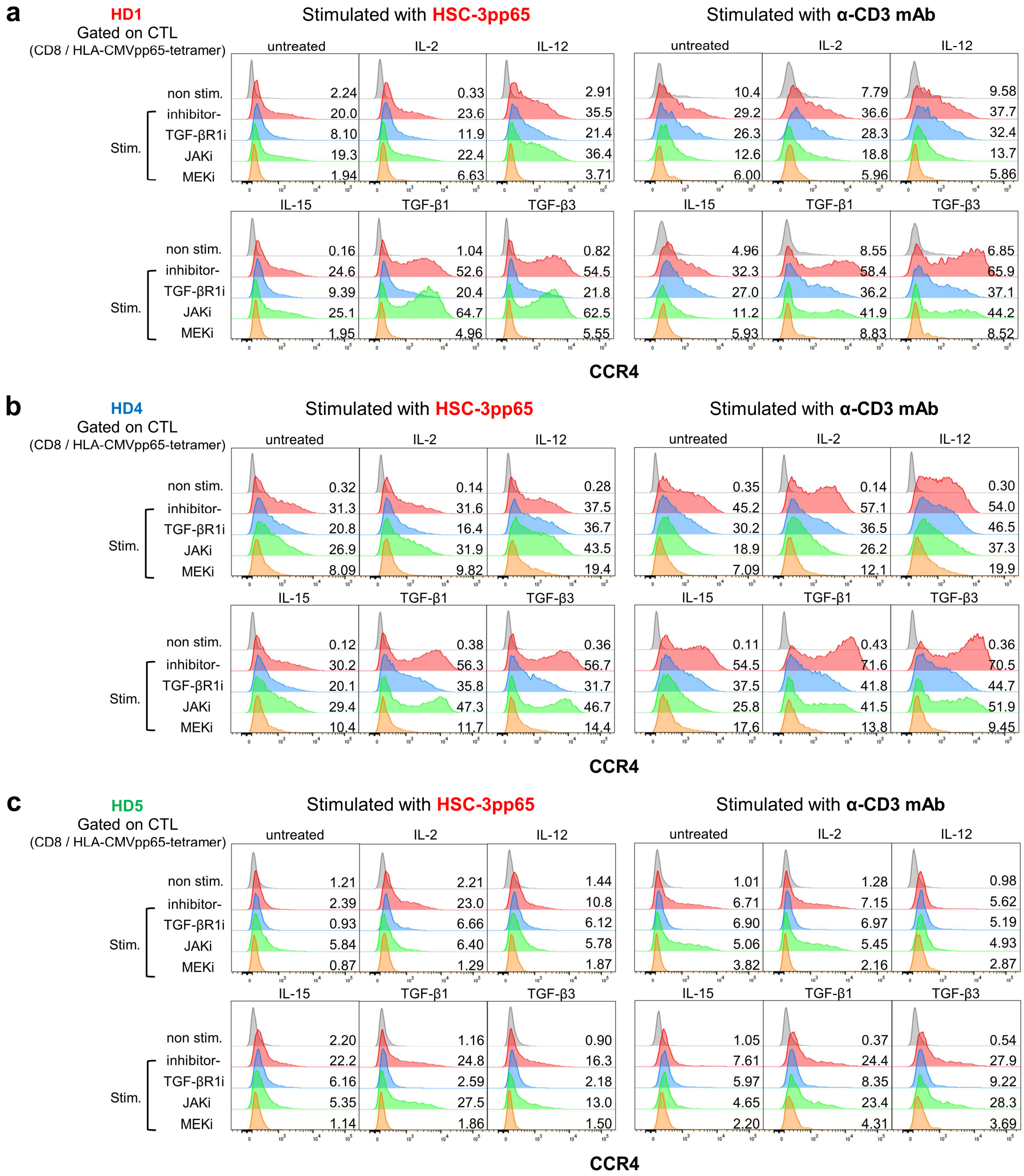

Supplementary Figure S3 The effects of TCR stimulation, cytokines, and inhibitors on CCR4 expression in CTLs. CMV-CTLs from three donors were stimulated with HSC-3pp65 or anti-CD3 mAb. Several cytokines (10 ng/mL) and kinase inhibitors (1 μmol/L) were added. CCR4 expression in CMV-CTL derived from (**a**) HD1, (**b**) HD4, and (**c**) HD5 on day2 are shown.

TCR: T-cell receptor, mAb: monoclonal antibody, non-stim.: non-stimulated, stim.: stimulated, TGF-βR1i: TGF-β receptor 1 inhibitor (SB525334), MEKi: MEK inhibitor (GSK1120212; trametinib), JAKi: JAK inhibitor (AZD1480)

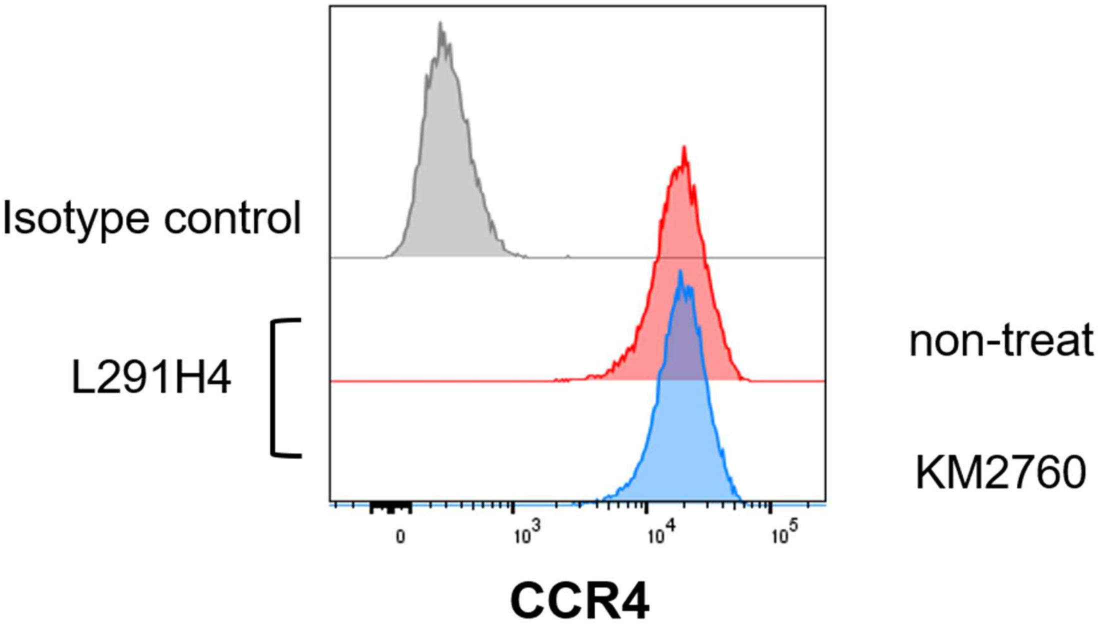

Supplementary Figure S4 KM2760 did not inhibit anti-CCR4 antibody clone L291H4 binding. MT-4 cells (adult T-cell leukemia/lymphoma cell line) were cultured in the presence or absence of 1μg/mL KM2760 for 1 h. Cells were stained with BV421 conjugated anti-CCR4 antibody clone-L291H4 and performed flowcytometry. Histogram of CCR4 expression is shown.

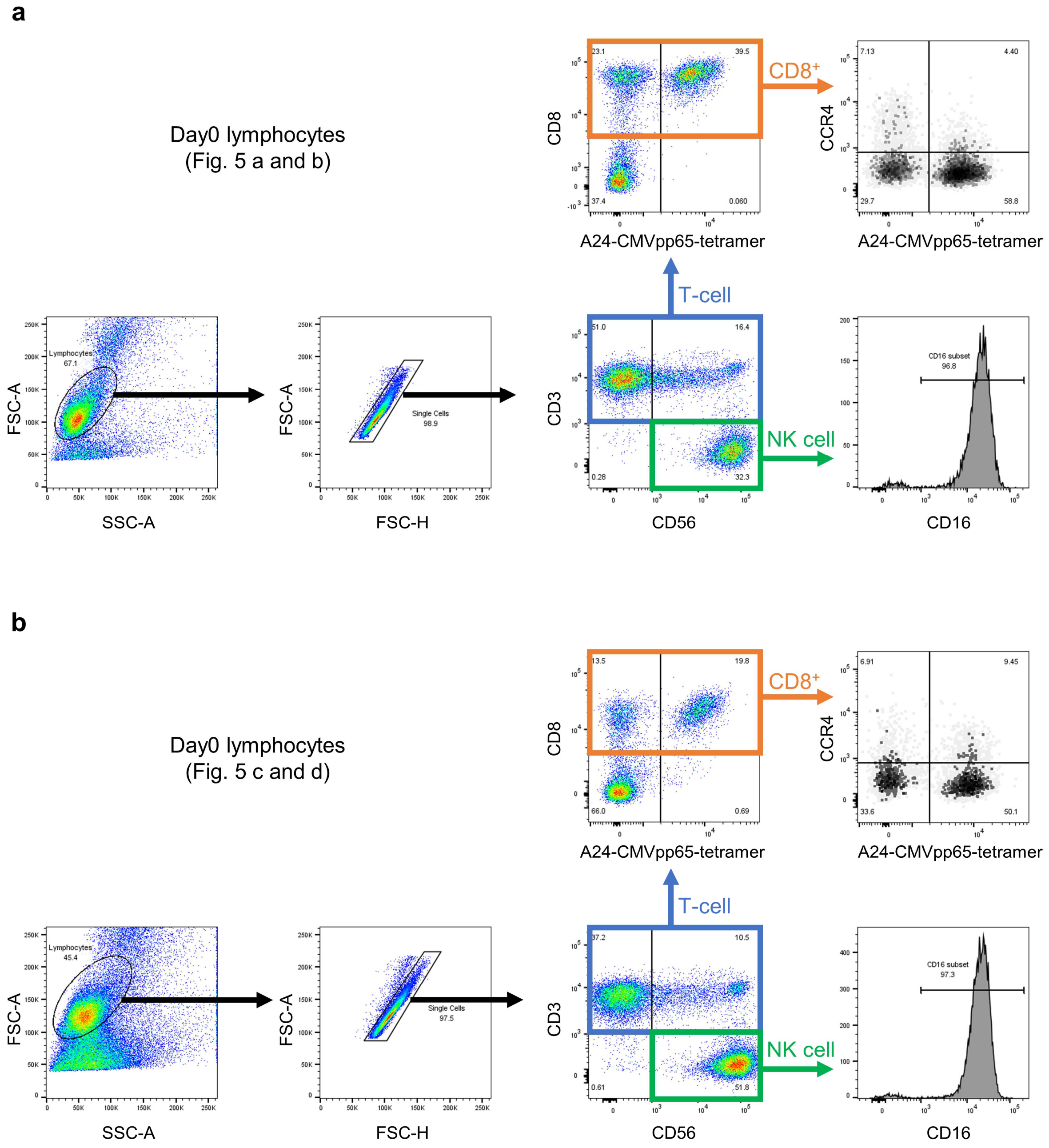

Supplementary Figure S5 Gating methods and cell subsets in day 0 lymphocytes used in effects of ADCC by KM2760 on the proliferation of CMV-CTLs. Gating methods and cell subsets correspond to the pre-treatment lymphocyte population used in the assessment of the effects of ADCC by KM2760 on the proliferation of CMV-CTLs are shown. (**a**) The lymphocyte population used in the experiment is shown in Fig. 5a, 5b and (**b**) in Fig. 5c, 5d

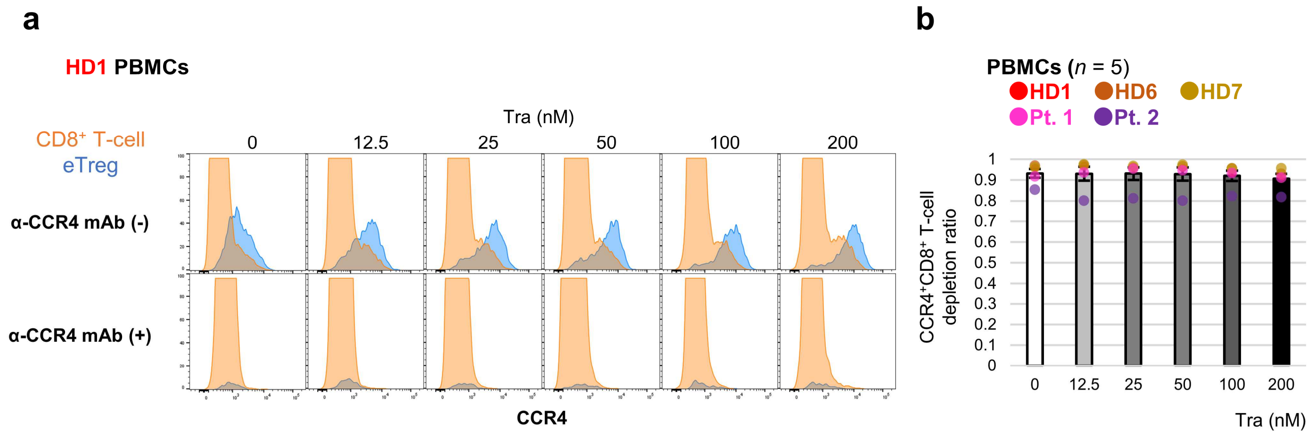

Supplementary Figure S6 Trametinib did not alleviate peripheral $CCR4^{+}CD8^{+}$ T-cell depletion. Analyses of the same samples as in Figure 6 are shown. PBMCs were incubated with 0.1 µg/mL KM2760 for 7 days, and flow cytometry was performed. $CD8^{+}$ T-cells were defined as $CD3^{+}CD8^{+}$, eTregs as $CD3^{+}CD4^{+}CD45RA^{-}$ $FoxP3^{high}$. The effects of KM2760 and trametinib on $CD8^{+}$ T-cells and eTregs are shown with a representative (**a**). The depletion rates of $CCR4^{+}CD8^{+}$ T-cells in the group with KM2760 are shown with a summary of result (*n* = 5, independent donors) (**b**). Error bars indicate standard errors

Supplementary information

scientific reports (submitted in 2022) –Ono et al.

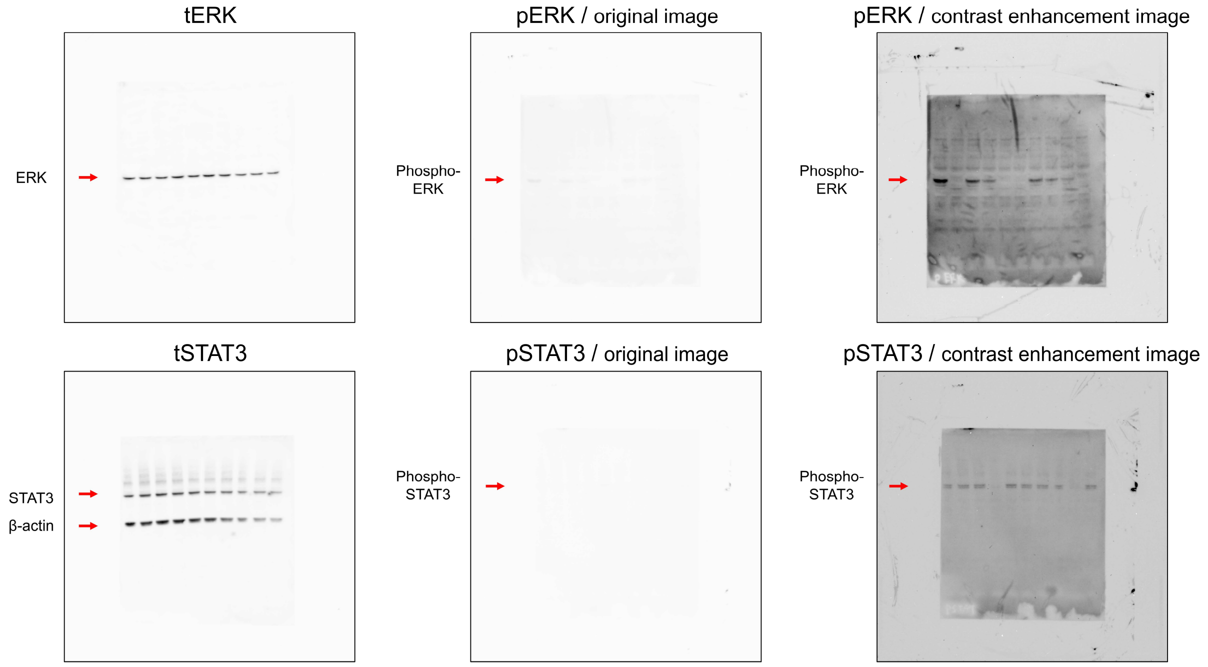

Supplementary Figure S7 Original images of the western blot is shown.
